# Development of a SYBR green I based RT-PCR assay for yellow fever virus: application in assessment of YFV infection in *Aedes aegypti*

**DOI:** 10.1186/1743-422X-9-27

**Published:** 2012-01-22

**Authors:** Paban Kumar Dash, Alain Boutonnier, Eric Prina, Shashi Sharma, Paul Reiter

**Affiliations:** 1 Institut Pasteur, Insects and Infectious Disease Unit, CNRS URA 3012, 25 rue du Docteur Roux, 75724 Paris, France; 2 Institut Pasteur, Immunophysiology and Intracellular Parasitism Unit, 25 rue du Docteur Roux, 75724 Paris, France; 3 Division of Virology, Defence R & D Establishment (DRDE), Jhansi Road, Gwalior, India

## Abstract

**Background:**

Yellow Fever virus (YFV) is an important arboviral pathogen in much of sub-Saharan Africa and the tropical Americas. It is the prototype member of the genus *Flavivirus *and is transmitted primarily by *Aedes (Stegomyia) *mosquitoes. The incidence of human infections in endemic areas has risen in recent years. Prompt and dependable identification of YFV is a critical component of response to suspect cases.

**Results:**

We developed a one-step SYBR Green I-based real-time quantitative RT-PCR (qRT-PCR) assay targeting the 5'NTR and capsid-gene junction--for rapid detection and quantification of YFV. The detection limit was 1 PFU/mL, 10-fold more sensitive than conventional RT-PCR, and there was no cross-reactivity with closely related flaviviruses or with alphaviruses. Viral load in samples was determined by standard curve plotted from cycle threshold (Ct) values and virus concentration. The efficacy of the assay in mosquitoes was assessed with spiked samples. The utility of the assay for screening of pooled mosquitoes was also confirmed. Replication of a Cameroon isolate of YFV in *Ae. aegypti *revealed a marked variation in susceptibility among different colonies at different days post infection (pi).

**Conclusions:**

The SYBR Green-1 based qRT-PCR assay is a faster, simpler, more sensitive and less expensive procedure for detection and quantification of YFV than other currently used methods.

## Background

Yellow fever (YF) is one of the most important arboviruses affecting humans in many tropical areas of sub-Saharan Africa and the Americas. According to the World Health Organization, there is a global annual incidence of 200,000 cases, including 30,000 deaths [1]. In the past decade, widespread recrudescence of YF has been attributed to deforestation, urbanization, declining herd immunity and climatic change [2]. In addition, imported cases of YF have been reported in travellers returning to United States and European countries from endemic countries [3,4].

The Yellow fever virus (YFV) is the prototype member of the genus *Flavivirus *in the family *Flaviviridae*. The genome of YFV is a linear, non segmented, positive-sense strand of RNA of approximately 11 kb. It encodes a single polyprotein that yields three structural (capsid, premembrane and envelope) and seven nonstructural (NS1, NS2A, NS2B, NS3, NS4A, NS4B and NS5) proteins [5].

The principal human-to-human vector of YFV is *Aedes (Stegomyia) aegypti*. The majority of YF infections are asymptomatic. Clinical cases range from a mild undifferentiated fever to severe illness characterised by jaundice, renal failure, hemorrhagic manifestations and shock [6]. The case-fatality rate in severe cases with hepato-renal dysfunction ranges from 20 to 50%. In spite of the availability of a safe vaccine, the prevalence and incidence of YF cases in enzootic areas in Africa and South America is increasing, but no specific antiviral therapy is still available [2]. In such circumstances, the early and specific detection can play a crucial role in the detection and management of cases.

At present, diagnosis of YF is routinely accomplished through conventional RT-PCR, immunoassay or by virus isolation. The genome based molecular assays are key to diagnosis in the acute phase of infection, before the appearance of antibody. The assays can also be used for detection of virus in field-caught mosquitoes [7]. The quantification of viral load has traditionally been performed by viral plaque assay, but quantitative RT-PCR (qRT-PCR) is now preferred, because of its simplicity, speed and sensitivity. It is also amenable to high-throughput screening and obviates the stringent requirement of biosafety laboratories [8]. A number of qRT-PCR assays have been described for detection and quantification of YFV. Most of these assays are based on TaqMan probe chemistry [9-12]. But SYBR Green chemistry offers a simpler and less expensive alternative whilst maintaining the advantages of qRT-PCR assays [13].

In the present study, we have developed a SYBR Green-I based one-step real-time RT-PCR assay targeting the 5' non-translated region (NTR) and capsid gene junction. The assay was optimized with orally infected *Ae. aegypti *mosquitoes and used to monitor the relative infectivity of a Cameroon isolate of YFV in different *Ae. aegypti *colonies.

## Results

### Standardization of SYBR green based real-time RT-PCR

**Figure 1:**
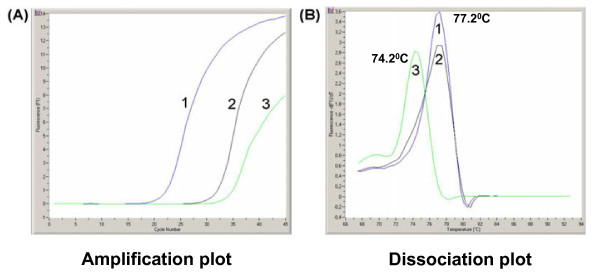
**YFV SYBR Green I based real-time RT-PCR showing the amplification and dissociation curve of three different samples; samples 1 and 2 correspond to YFV specific amplicon, sample 3 corresponds to non specific amplicon (A) Amplification plot and (B) Dissociation plot through melting curve analysis of the amplicon**.

Primer sets for the assay were shortlisted based on the sequence-alignment of different YFV and other closely related flavivirus genomes available from the National Center for Biotechnology Information database http://www.ncbi.nlm.nih.gov. Finally, a set of published primers targeting the conserved 5' NTR and capsid gene junction of YFV was selected on the basis of their specificity, relative conservation across strains, primer compatibility and assay efficiency [9]. The thermal profile of the qRT-PCR assay and the concentration of primers were optimized to increase the sensitivity and specificity of the assay. Optimal assay condition was established at an annealing temperature of 58°C using 500 nmol of each of the primers (YFS and YFAS) in a 10 μL of reaction scale. The melting curve analysis revealed the YFV-specific amplicon in this assay melts at 77.2°C. The non-specific amplification seen in some reactions was found to melt at 74.2°C (Figure 1). The analysis of result in amplification plot is studied in conjunction with melting curve.

### Sensitivity of real-time RT-PCR and conventional RT-PCR

**Figure 2:**
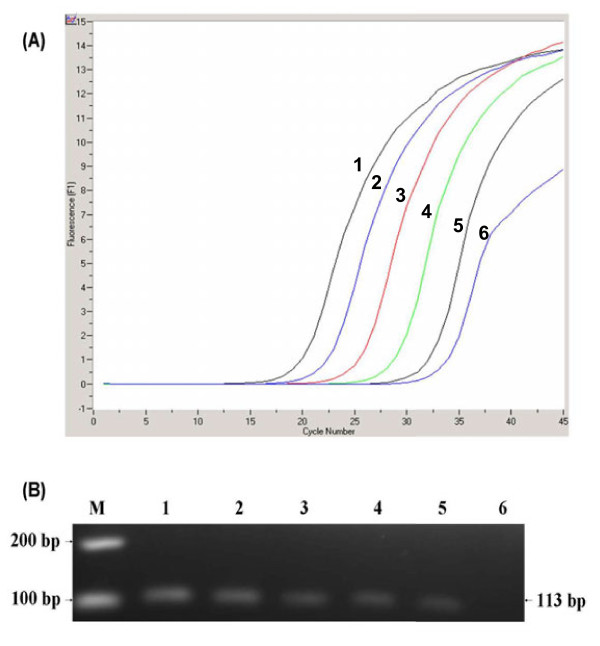
**Comparative sensitivity of SYBR Green I real-time RT-PCR assay vs. conventional RT-PCR**. (A) Sensitivity of YFV RT-PCR assay as shown in the amplification plot (curves from left to right corresponds to decreasing concentration of YFV from 10^5^ to 1 PFU/mL). The detection limit for the assay was 1 PFU/mL. (B) Sensitivity of conventional RT-PCR as observed by 113 bp amplicon on 2% agarose gel with a detection limit of 10 PFU/mL. Lane M: 100 bp DNA ladder (Fermentas, USA); lanes from 1 to 6 corresponds to decreasing concentration of YFV from 10^5^ to 1 PFU/mL.

**Figure 3:**
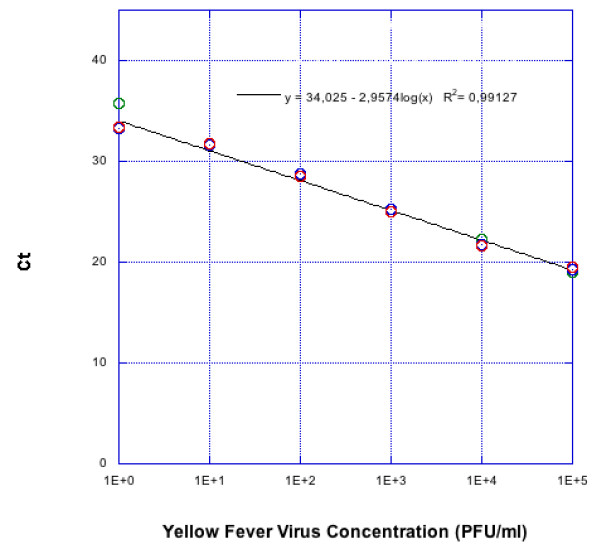
**Standard curve of YFV-specific SYBR Green Real-time RT-PCR used for the quantification of viral load in test samples, generated from the Ct values obtained against 10 fold serial dilutions of known concentration of YFV RNA in triplicate**. The coefficient of determination (R^2^) and slope of the regression curve are indicated.

The minimum detection limit of the real-time RT-PCR assay was 1 PFU/mL, with linearity over a wide dynamic range of log 5-0 PFU/mL. The coefficient of determination (R^2^) of the standard curve was 0.99 with a slope value of 2.95. The same 10 fold serially diluted RNA was also evaluated by conventional RT-PCR to compare sensitivity; the minimum detection limit was 10 PFU/mL (Figures 2 and 3).

The minimum detection limit of qRT-PCR and conventional RT-PCR was 30 and 300 YFV RNA copies respectively, as determined using serially diluted in vitro transcribed RNA. The qRT-PCR assay was linear over a 7 log range from 3 × 10^7^-3 × 10^1 ^YFV RNA copies per reaction Additional file 1: Figure S1.

### Specificity

The assay was highly specific for YFV; no cross-reaction was observed with the other seven closely related flaviviruses (DENV 1-4, JEV, WNV, SLEV) nor with the alphavirus CHIKV and there was no false positivity when tested against uninfected *Ae. aegypti *and *Ae. albopictus *mosquitoes. The nucleotide sequencing of the YFV positive amplicon revealed sequences corresponding to the 5'NTR-capsid genomic junction of YFV genome.

### Infection of *Aedes aegypti*

The YFV qRT-PCR assay was found to be highly sensitive with a detection limit of 1 PFU/mL in YFV spiked *Aedes aegypti*. The assay was also found non reactive against *Aedes aegypti* spiked with DENV 1-4, JEV, WNV, SLEV and CHIKV.

**Figure 4:**
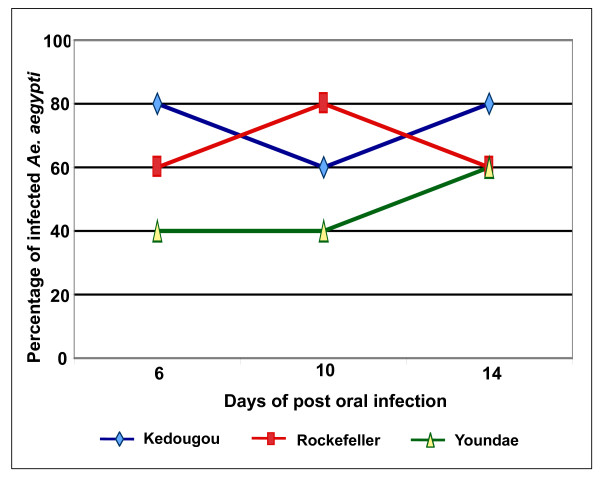
**Infection rates of different colonies of *Aedes aegypti *orally fed with YFV (Cameroon strain) over extrinsic incubation period**. Day corresponds to the screening of mosquitoes following infected blood meal feeding.

The infection rate of YFV in three Aedes colonies was monitored at day 6, 10 and 14 post infection (pi). Wide variation in infection rate was observed both in individual mosquitoes as well as among colonies (Figure 4). The Kedougou strain showed a higher infection rate of 80% compared to others on day 14 pi. The titre in individual mosquitoes varied greatly with a high of 2 × 10^3 ^PFU/mL on day 14 pi (detail result not shown).

**Figure 5:**
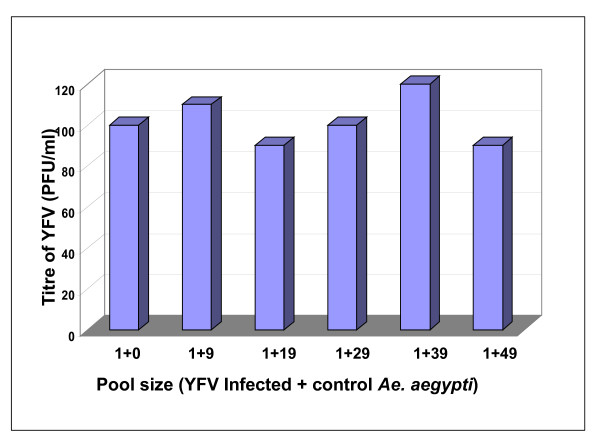
**Effect of increasing pool size of *Ae. aegypti *mosquitoes on detection and quantification of yellow fever virus by YFV specific SYBR Green Real-time RT-PCR**. The mean titre of pools was found to be 101 ± 12 PFU/mL.

Further, The YFV qRT-PCR assay was used to monitor the detection efficiency of YFV in pooled mosquitoes. The addition of one YFV infected mosquito to multiple uninfected mosquitoes (0 to 49) did not affect the detection efficiency. The mean titre of pools was found to be 101 ± 12 PFU/mL (Figure 5).

## Discussion

There is a real and present danger of catastrophic Yellow fever epidemic transmission between humans if the virus were introduced into the urban environment. For this reason, there is a need for a rapid and sensitive technique for detecting the Yellow fever virus with high specificity. Molecular assays based on RT-PCR are now utilized for specific and sensitive detection of arboviruses. qRT-PCR is widely considered an efficient tool for detection of viruses due to its speed and reproducibility [14]. qRT-PCR has also been successfully applied for the study of several arbovirus-vector interaction [7,11,15,16].

The problem associated with false negative test results with probe based assays prompted us to develop a SYBR Green I based qRT-PCR assay. The false negative results are primarily attributed to mutation in the target sequence of the probe [13,17,18]. Thus, Whiley and Sloots reported non-detection of a strain of Respiratory Syncytial Virus (RSV) in a TaqMan probe-based assay that resulted from a single nucleotide mismatch [17], and the same limitation has been observed in a TaqMan-based assay for detection of West Nile virus [13]. More recently, an LNA (locked nucleic acid) probe-based assay failed to detect a Brazilian YFV isolate, also due to a single transversion (G-A) in the target sequence of the probe [18]. Moreover, detailed analysis of YFV genome database at NCBI revealed presence of similar transversions (G-T) in the probe target region (reported by Drosten et al., 2002) of five YFV sequences (GenBank Accession no. DQ235229, AY968065, U52422, U52395, U52392). The probe independent SYBR Green-I based qRT-PCR assay has the potential to detect such mutant strains [13]. YFV being a RNA virus is prone to emergence of novel mutant strains, which warrants for development of sensitive probe independent assays.

The SYBR Green based qRT-PCR that we have developed provides a simple and economic alternative for high throughput screening, with the important advantage that the whole process of RT and qPCR can be completed within 1 hour. The analysis of amplification plot in conjunction with melting curve, as practised in this study has resulted in discriminating non-specific amplification. This is recommended in all SYBR Green based Real-time RT-PCR assay to avoid false positive results, since SYBR Green can also intercalate into non-specific DNA including primer-dimer [13]. The assay was found to be 10-fold more sensitive compared to conventional RT-PCR, which confirms its utility in detecting low viremic samples (we are using this as a model system for general studies of infection in certain mosquito organs). The quantitation of the viral load in samples was carried out using a standard curve obtained by plotting cycle threshold (Ct) values versus known virus concentration. The detection limit of this assay was found to be 1 PFU/mL, (30 RNA copies/reaction; equivalent to 3000 RNA copies/ml), similar to other TaqMan based assays [9-12]. A comparison of real-time RT-PCR with viral plaque assay revealed a significant correlation between YFV genome to infectious particle, which varied from 1000 to 5000:1 [10]. Similar 3-log ratio difference was also reported for DENV-2 [19]. The specificity of the assay was established through melting curve analysis by differential melting temperature (Tm). This aids in differentiating non specific amplifications. The specificity of this assay was also verified by including a battery of closely related flaviviruses (DENV 1 through 4, JEV, WNV, SLEV) and alphavirus (CHIKV). The non reactivity with these related viruses confirm the utility of this assay in YFV endemic areas, where these related viruses are also co-circulating. Further, the analysis of nucleotide sequence of the amplicon in YFV positive samples confirmed the specificity of the assay.

The qRT-PCR assay was subsequently applied to monitor the infection rate of YFV in three Ae. aegypti mosquito colonies during the extrinsic incubation period. Variation in infection rate was clearly discernable among different Aedes colonies and among individual mosquitoes. Variation in infection rate was also noticed at different days of post infection. The fluctuation in infection rate at different days of extrinsic incubation period was also reported for DENV and CHIKV [7,19,20] This may be attributed to the differential susceptibility of the different mosquito populations. The fluctuation in infection rate and viremia on different days has been attributed to a range of factors including digestion of blood meal at early stage to decline in nutrient availability in later stage [7,19].

Its utility for screening of YFV infected mosquito pools was further confirmed from that fact that there was neither inhibition of detection nor with quantification with addition of up to 49 uninfected mosquitoes to one infected mosquito. The detection of a single infected mosquito in a pool size up to 50 makes this a reliable assay for surveillance of YFV.

## Conclusions

This study clearly demonstrated the potential usefulness of SYBR Green-I based qRT-PCR assay for detection and quantification of YFV and for studying the replication of YFV virus. As a powerful tool of viral quantification even in a single mosquito, this assay can be useful for studying vector competence and transmission of YFV in potential vectors from endemic and non-endemic areas.

## Methods

### Virus

We used an isolate of YFV obtained from a human case in Cameroon in 1990 and maintained in *Ae. albopictus *clone C6/36 cells (first at Institut Pasteur, Dakar, and then at Institut Pasteur, Paris) [21]. Infectivity titer of the virus was determined by plaque assay in Vero cells following standard protocol [22]. Seven closely related flaviviruses--West Nile virus (WNV, Israel 98), Dengue virus serotypes (DEN-1, Hawaii; DEN-2, ThNH7/93; DEN-3, PhMH-J1-97; DEN-4, SLMC 318), Japanese encephalitis virus (JEV, JaOArS982) and St. Louis encephalitis virus (SLEV, Parton) were used for cross-reactivity assay, as well as an Alphavirus, Chikungunya virus (CHIKV, DRDE-07). The West Nile virus strain was propagated at Institut Pasteur, Paris, the remainder in C6/36 cells at the Virology Division, DRDE, Gwalior, India.

### Mosquitoes

Three different laboratory-reared *Ae. aegypti *strains (Rockefeller, Kedougou and Youndae) were employed in the present study to monitor the infection of YFV during extrinsic incubation period. The mosquitoes were maintained at 26°C, with a 14:10 Light:Dark cycle. Adults were maintained with 10% sucrose *ad libitum*.

### Isolation of viral RNA

Viral RNA was extracted from 100 μL of supernatant of YFV- and WNV-infected culture and from individual YFV-infected mosquitoes using NucleoSpin RNA II kit (Macherey-Nagel, France), following the manufacturer's protocols. The individual mosquitoes were homogenized in 2 mL tubes with Leiboviz 15 medium and glass beads using TissueLyser II system (Qiagen, Germany) and the clarified homogenate were used for extraction of RNA. Viral RNA from supernatant of DENV 1-4, JEV, SLEV and CHIKV-infected culture was extracted using QIAamp viral RNA mini kit (Qiagen, Germany), according to the manufacturer's protocols. It was eluted in 50 μL of nuclease-free water and stored at -70°C.

### Confirmation of RNA by RT-PCR

Supernatant from infected cultures was assayed by conventional RT-PCR to confirm the presence of specific viral RNA [23-26].

### Selection of primers

A number of self-designed and published oligonucleotide primer sets for YFV were evaluated to standardise the qRT-PCR protocol. Finally, a set of primers YFS: AATCGAGTTGCTAGGCAATAAACAC (genomic position: 29-53) and YFAS: TCCCTGAGCTTTACGACCAGA (genomic position: 141-121) targeting the 5' NTR and capsid gene junction of YFV [9] were selected for the study.

### SYBR green-I based real-time RT-PCR

SYBR Green I-based one-step real-time quantitative RT-PCR amplification was performed in a LightCycler Carousel-based system (Roche, USA). Initially the assay was optimized with YFV positive RNA using 'LightCycler RNA Master SYBR Green I kit' (Roche, Germany). Briefly, samples were assayed in a 10 μL reaction mixtures containing 3.75 μL of 2.7 × SYBR Green reaction mix, 1 μL of RNA and a final concentration of 500 nM of each YFS and YFAS primer, and 3.25 mM Mn(acetate) in a LightCycler capillary. The thermal profile comprised of 20 min of reverse transcription at 61°C one cycle and 90 sec of denaturation at 95°C, followed by 4 cycles of PCR amplification at 95°C for 5 sec, 58°C for 15 sec, and 72°C for 15 sec. Following amplification, a melting curve analysis programme was performed to verify the authenticity of the amplified products by their specific melting temperatures (Tm) according to the instructions of the manufacturer (Roche, Germany). The resulting products were subjected to nucleotide sequencing to confirm their specificity.

### Determination of detection limit and construction of standard curve

The detection limit of the assay system was determined by mosquito spiked with 10-fold serial dilutions of RNA extracted from plaque-quantified YFV in triplicate. The Ct values obtained against known concentrations of serially diluted YFV were used to construct standard curve. Results were also compared with the conventional RT-PCR.

In addition, the detection limit was also determined in terms of RNA copy numbers. The YFV RNA standard was produced using T7 transcription kit (MBI Fermentas, USA) following the protocol standardized in our laboratory [26]. The detection limit of both qRT-PCR and conventional RT-PCR assay was determined from mosquito spiked with 10-fold serial dilutions of 3 × 10^8 ^copies of YFV RNA transcripts.

### Conventional RT-PCR

Conventional RT-PCR was performed with the same primer sets (YFS and YFAS) targeting the 113 bp of the YFV genome to compare the sensitivity of real-time RT-PCR assay. Amplification was carried out in 25 μL total reaction volume by using Titan One Tube RT-PCR kit (Roche, Germany) as per the manufacturer's protocol. After amplification, the PCR products were electrophoresed on a 2% agarose gel and visualized on a Gel Documentation system (Bio-Rad, USA).

### Oral infection of mosquitoes

Seven-day old female *Ae. aegypti *mosquitoes were deprived of sucrose solution 24 h prior to feeding. The mosquitoes were offered an infected blood meal at 37°C for 30 min from water-jacketed membrane feeders via a Hemotek membrane feeding system (Discovery Workshops, UK). The infected meal was composed of washed rabbit erythrocytes (67%) in YFV culture supernatent (33%). Adenosine triphosphate (ATP), a phagostimulant, was added at a final concentration of 5 mM. The infected bloodmeal had an YFV titre of 2 × 10^5 ^plaque-forming units (PFU)/mL. A sample of pre-feeding and post-feeding blood meal was collected and stored at -80°C for later titration. After feeding, mosquitoes were cold-anesthetized and pools of ten fully-engorged insects removed, transferred to small cardboard containers, and maintained with 10% sucrose *ad libitum *at 28°C. Samples were collected at 6, 10 and 14th day of post infection and stored at -80°C. All the samples were screened by qRT-PCR to determine their infection rate. Oral infection and subsequent manipulations of infected mosquitoes were carried out in a biosafety level 3 (BSL-3) insectary.

To check the effectiveness of the qRT-PCR to screen mosquito pools, one engorged individual *Aedes *(infected with 6 × 10^3 ^PFU/mL) was added to multiple uninfected mosquitoes (9, 19, 29, 39 and 49), before extraction of RNA.

## Competing interests

The authors declare that they have no competing interests.

## Authors' contributions

PKD standardized the qRT-PCR assay, performed plaque assay and wrote the MS. AB performed virus propagation and mosquito infection experiments. EP helped in qRT-PCR assay. SS has performed cross reactivity studies. PR participated in project conception, experimental design and writing of the MS. All authors read and approved the final manuscript.

## Supplementary Material

Additional file 1 **Figure S1**. A. Standard curve of YFV-specific SYBR Green Real-time RT-PCR, generated from the Ct values obtained against 10 fold serial dilutions of known concentration of YFV in vitro transcribed RNA. The coefficient of determination (R^2^) and slope of the regression curve are indicated. B. Comparative sensitivity of SYBR Green I real-time RT-PCR assay vs. conventional RT-PCR. Sensitivity of YFV RT-PCR assay as shown in the amplification plot (curves from left to right correspond to decreasing concentration of YFV in vitro transcribed RNA from 3 × 10^8 ^to 3 RNA copies). The detection limit for the assay was 30 RNA copy. (B) Sensitivity of conventional RT-PCR as observed by 113 bp amplicon on 2% agarose gel with a detection limit of 300 RNA copy. Lane M: 100 bp DNA ladder (Fermentas, USA); lanes from 1 to 6 corresponds to decreasing concentration of YFV from 3 × 10^8 ^to 3 RNA copies. Click here for file
