# Supplementary material for: Development of a SYBR green I based RT-PCR assay for yellow fever virus: application in assessment of YFV infection in Aedes aegypti
Source: Virol J. 2012 Jan 22;9:27. doi: 10.1186/1743-422X-9-27 (PMC3296605; doi:10.1186/1743-422X-9-27)
Supplement: Additional file 1 — Figure S1. A. Standard curve of YFV-specific SYBR Green Real-time RT-PCR, generated from the Ct values obtained against 10 fold serial dilutions of known concentration of YFV in vitro transcribed RNA. The coefficient of determination (R2) and slope of the regression curve are indicated. B. Comparative sensitivity of SYBR Green I real-time RT-PCR assay vs. conventional RT-PCR. Sensitivity of YFV RT-PCR assay as shown in the amplification plot (curves from left to right correspond to decreasing concentration of YFV in vitro transcribed RNA from 3 × 10^8^ to 3 RNA copies). The detection limit for the assay was 30 RNA copy. (B) Sensitivity of conventional RT-PCR as observed by 113 bp amplicon on 2% agarose gel with a detection limit of 300 RNA copy. Lane M: 100 bp DNA ladder (Fermentas, USA); lanes from 1 to 6 corresponds to decreasing concentration of YFV from 3 × 10^8^ to 3 RNA copies. [file 1743-422X-9-27-S1.PPT]

## Slide 1
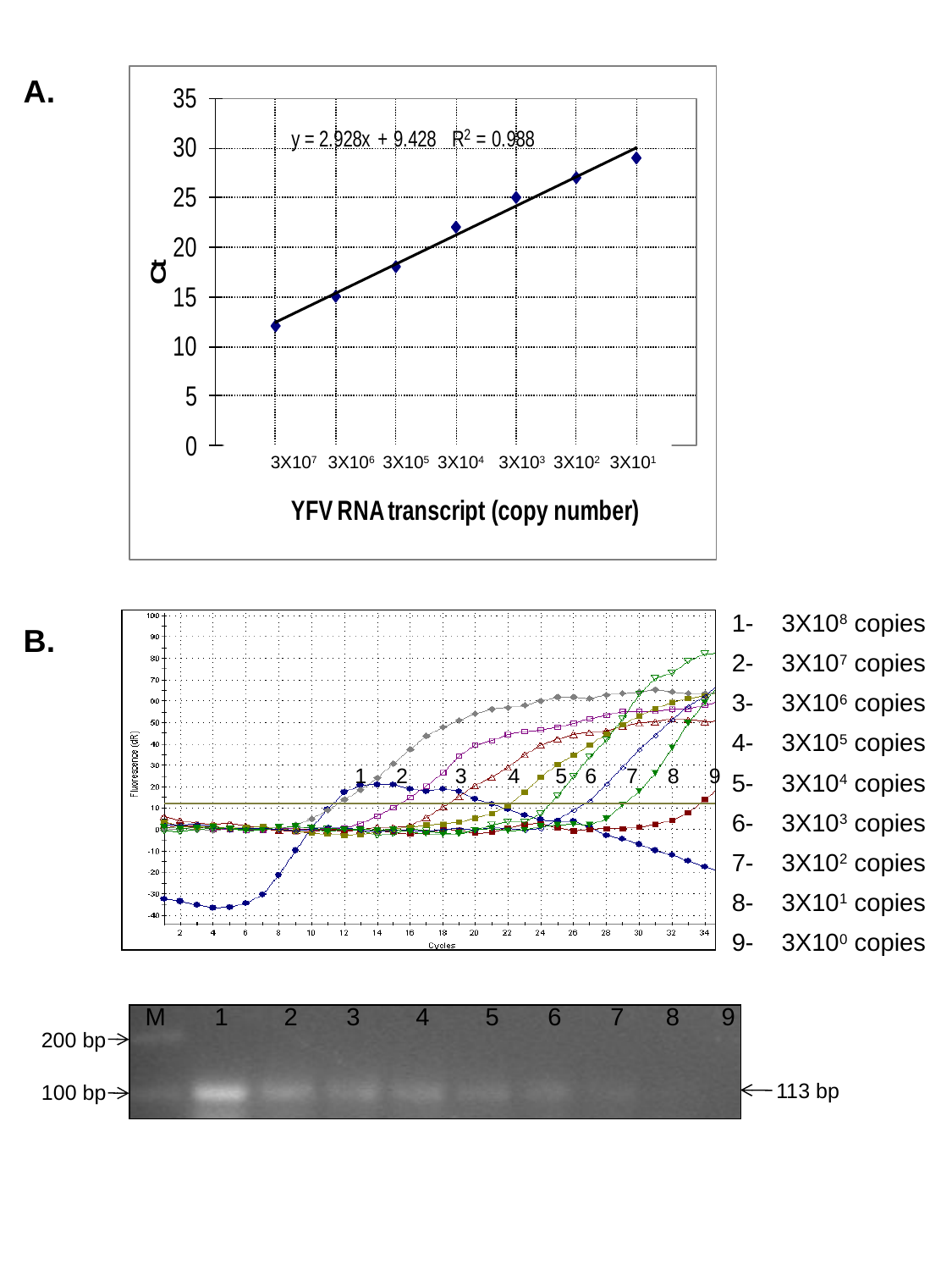

A.
 3X107 3X106 3X105 3X104 3X103 3X102 3X101
1- 3X108 copies
2- 3X107 copies
3- 3X106 copies
4- 3X105 copies
5- 3X104 copies
6- 3X103 copies
7- 3X102 copies
8- 3X101 copies
9- 3X100 copies
B.
M 1 2 3 4 5 6 7 8 9
200 bp
113 bp
100 bp
1 2 3 4 5 6 7 8 9
